# Nurses’ Compliance with Delirium Assessment Protocol in a Tertiary Hospital: A Retrospective Study

**DOI:** 10.3390/healthcare14060704

**Published:** 2026-03-10

**Authors:** Siti Nur I’faaf Binte Mohd Amin, Siew Hoon Lim, Min Yi Tan, Tau Ming Liew

**Affiliations:** 1 Deapartment of Nursing, Singapore General Hospital, Outram Road, Singapore 169608, Singapore; lim.siew.hoon1@sgh.com.sg (S.H.L.); tan.min.yi@sgh.com.sg (M.Y.T.); 2 Deparment of Psychiatry, Singapore General Hospital, Outram Road, Singapore 169608, Singapore; liew.tau.ming@singhealth.com.sg; 3 Singhealth Duke-Nus Medicine Academic Clinical Programme, Duke-NUS Medical School, 8 College Road, Singapore 169857, Singapore; 4 Health Services Research and Population Health, Duke-NUS Medical School, 8 College Road, Singapore 169857, Singapore; 5 Saw Swee Hock School of Public Health, National University of Singapore, 12 Science Drive 2, #10-01, Singapore 117549, Singapore

**Keywords:** 4AT, delirium, nursing, compliance, timeliness

## Abstract

**Highlights:**

**What are the main findings?**

- Suboptimal compliance to 4AT screening assessment.
- Delayed screening of delirium among patients.

**What are the implications of the main findings?**

- Further quality improvement and research study is needed to improve nurses adherence to assessment protocol.
- Hospitals should focus on improving timeliness of performing 4AT.

**Abstract:**

Background/Aim: Delirium is an acute neurocognitive disorder commonly affecting older adults. With Singapore’s aging population, the early detection of delirium has become increasingly critical. The 4 ‘A’s test (arousal, attention, abbreviated mental test, acute change) (4AT) assessment is a validated screening tool designed to detect delirium and is widely used in older adults. Despite its routine application, both compliance and timeliness with the Modified 4AT protocol have not yet been assessed in an acute tertiary care setting in Singapore. Delayed or inconsistent use may lead to late diagnosis and management. Therefore, this study aims to assess the compliance rate and timeliness of the Modified 4AT when screening for delirium among older adult inpatients in an acute tertiary hospital in Singapore. Methods: For this retrospective study, electronic medical records of patients aged 65 and above admitted in an acute local tertiary hospital in Singapore between 1 June 2022 and 30 June 2023 were included in the analysis. Data extraction and record reviews were conducted between September 2023 and June 2025 using the hospital’s electronic medical record system. This study was submitted to the institution’s review board but received an exemption due to its nature as a retrospective review without patient contact (CIRB Ref: 2023/2457). Results: Of the 4821 admissions, 73.5% (*n* = 3545) received an assessment using a 4AT. Among those assessed, only 32.9% (*n* = 1168) underwent screening within the recommended 24 h of admission in accordance with the hospital’s Nursing Operational Guidelines. The majority (*n* = 2377) had their 4AT assessments done after 24 h. Conclusions: Preliminary findings suggest suboptimal compliance with delays in Modified 4AT assessments in the acute setting, potentially affecting patient outcomes. Strengthening adherence to timely delirium screening protocols may improve the care quality for older adults who are at risk.

## 1. Introduction

As defined by the Diagnostic and Statistical Manual of Mental Disorders (DSM-5), delirium is an acute neuropsychiatric syndrome characterized by sudden and unpredictable structural and cognitive changes. It is accompanied by disturbance to attention and awareness which fluctuates in severity throughout the day, coupled with cognitive disturbance such as memory, orientation, language, visuospatial ability, or perception deficits [1]. These disturbances should not be explained by a pre-existing disorder, nor should it occur in the context of severely reduced arousal, such as coma. Additionally, there must be evidence pointing these disturbances towards direct physiological consequence of an underlying medical condition, substance intoxication or withdrawal, or exposure to a toxin, or is due to multiple etiologies [1]. Older patients are at significantly higher risk for developing delirium [2]. As people age, the brain gradually has lesser functional capacity to compensate for increases in metabolic requirements such as those caused by illness or stressors [3]. This makes older adults more susceptible to delirium [3]. As Singapore faces a demographic shift with a growing proportion of older individuals [4], delirium is anticipated to become a major concern for healthcare systems.

A systematic review and meta-analysis of 35 studies involving 12,097 participants reported a 23.7% pooled prevalence and 13.5% pooled incidence of delirium in medically hospitalized older patients [5]. Undiagnosed or delayed delirium assessments during hospital admissions can lead to complications such as longer hospital stays, increased mortality, and poor long-term functional recovery [6,7,8,9,10]. Routine and timely implementations of delirium risk assessments are key to improving patient outcomes and enhancing the overall quality of care for older adults [6,11]. The 4 ‘A’s test (arousal, attention, abbreviated mental test, acute change) (4AT) assessment is a widely utilized and validated screening tool designed to detect delirium [12,13]. The tool consists of four components: alertness, the abbreviated mental test (AMT), attention, and the identification of any acute changes or fluctuations in cognitive function [12]. According to MacLullich et al. (2019) [12], there has been widespread adoption and integration of 4AT in clinical practice and policy globally. This highlights 4AT credibility as a trusted and validated tool with broad applicability within hospital settings. However, despite the strengths of 4AT tool, poor compliance with performing the assessment appears to be a key limitation. Studies have shown variability in compliance and timeliness when performing 4AT assessment that can delay delirium detection and compromise patient outcomes [13].

This study aims to report on the compliance and timeliness of delirium assessment protocol among a cohort of hospitalized patients in an acute local tertiary hospital in Singapore.

This study aims:

1. To determine the compliance rate of delirium risk assessment among patients admitted between June 2022 and June 2023.
2. To examine the timeliness of delirium risk assessment among the study cohort.
3. To identify clinical and demographic characteristics among patients associated with high delirium risk.

## 2. Materials and Methods

### 2.1. Study Design and Study Population

This was a retrospective descriptive audit study conducted in an acute tertiary hospital in Singapore.

The electronic medical records of patients aged 65 and above and admitted to acute ward settings between 1 June 2022 and 30 June 2023 were included for analysis. Patients admitted to intensive care units, day surgery, discharge lounges, and community hospitals were excluded. A summary of the detailed eligibility criteria is in Table 1.

### 2.2. Data Collection Process

Electronic medical records of all patients admitted between 1 June 2022 and 30 June 2023 were extracted exclusively by the hospital’s informatics team not involved in the study. As the aim was not to assess longitudinal change and no major changes in 4AT policy or workflow occurred during the study period, a period of one year was chosen. The extracted dataset was subsequently transferred to an independent third-party representative for data anonymization. Following anonymization, the third party randomly selected 10,000 patient records using an internal random number generator and released the anonymized dataset to the research team. A dataset of 10,000 patient records was chosen as it remained in accordance with the predefined extraction limits of the institutional data request system. The merged and anonymized datasets were sent back via several password-locked files. The research team then screened the 10,000 records for eligibility based on the inclusion criteria. After applying the eligibility criteria, a total of 4821 patient records were included in the final analysis.

### 2.3. Study Variables

Included study variables used in this study were patient demographics, clinical variables, and outcomes. Demographics included age (years), sex (male/female), and ethnicity (Chinese, Malay, others). Clinical variables included admission ward, activity assessment (bedfast, chairfast, walks frequently, walks occasionally, blanks), presence of indwelling urinary catheter (yes, no/blanks), length of stay (≤5 days, 6–10 days, 11–15 days, >15 days), Malnutrition Universal Screening Tool score (0, 1, ≥2, blanks), and physical restraint use (yes/no). Delirium screening variables included 4AT completion (≤24 h, >24 h) and 4AT score (≥4 or <4).

### 2.4. Instruments

#### 2.4.1. 4AT Delirium Screening Tool

The 4AT assessment tool has been used as one of the cognitive screening assessment tools to detect delirium in acute local tertiary hospitals in Singapore. The tool consists of four components: alertness, the abbreviated mental test (AMT), attention, and acute changes or fluctuations [12,14]. 4AT scores range from 0 to 12, with scores of ≥4 indicating a potential delirium diagnosis, 1–3 suggesting cognitive impairment but not delirium, and score of 0 suggestive of no delirium and no moderate–severe cognitive impairment [14].

According to the hospital’s Nursing Operational Guidelines, an assessment using the 4AT tool should be performed upon a patient’s admission and on a subsequent daily basis. Registered nurses should use the tool in delirium screening for all inpatients who are more than 65 years old upon admission, or when a patient is less than 65 years old but presents with an acute confusion state that cannot be attributed to any gross pathology or substance abuse. If a patient scores 4 or above (for the first time), the medical team will be alerted. Subsequent screening results with scores of 4 and above can serve as a means to monitor the patient’s progress. Based on the screening results, registered nurses will then carry out non-pharmacological interventions according to hospital policy or the delirium care bundle as appropriate.

#### 2.4.2. Malnutrition Screening Tool

Malnutrition Universal Screening Tool (MUST) is a validated screening instrument that can be used to assess malnutrition risk among the cohort of patients [15]. The tool comprises three components: body mass index, unintentional weight loss, and acute disease effect. MUST scores range from 0 to ≥2, with scores of 0 indicating low risk, 1 medium risk, and ≥2 high risk of malnutrition.

### 2.5. Ethical and Legal Considerations

This study was submitted to the institution’s review board but received an exemption due to its nature as a retrospective review without patient contact (CIRB Ref: 2023/2457).

The de-identification of datasets was carried out by a third party not involved in this study.

### 2.6. Statistical Analysis

We calculated both the compliance rate and the time to the first screening. A case was counted as compliant if the first 4AT was documented within 24 h. A 4AT assessment documented more than 24 h after a patient’s admission was considered and counted as one instance of non-compliance. The total compliant counts divided by the total number of patients (in percentages) were used to determine the overall compliance rate in the delirium risk assessment protocol among a cohort of hospitalized patients. Descriptive statistics were used to summarize the results using percentages (%). If no 4AT score was documented, the assessment was considered as not completed.

This study used Statistical Package for Social Sciences 26.0 software to compare differences between categorical variable subgroups (e.g., sex, ethnicity, catheter use, restraint use, and malnutrition risk). A chi-square test was performed to determine whether there was a significant association between the variable subgroups. Given the nature of this study as descriptive, analyses were limited to unadjusted comparisons. The significance level was set at *p* < 0.05.

## 3. Results

A total of 10,000 admissions were randomly extracted from the electronic medical record. After applying eligibility criteria, 4821 cases met inclusion criteria and were included in the final analysis. As the initial sampling was random and no further selection was applied, the final cohort was likely representative of eligible patients within the hospital during the study period. Data were extracted using a standardized data collection form. The following information was retrieved for each patient who scored 4AT ≥ 4: (1) age; (2) gender; (3) ethnicity; (4) total 4AT scores; (5) use of indwelling catheter (IDC); (6) restraint use; (7) activity assessment; (8) length of stay (LOS); and (9) Malnutrition Universal Screening Tool scores.

### 3.1. Compliance and Timeliness with 4AT Screening

**Figure 1:**
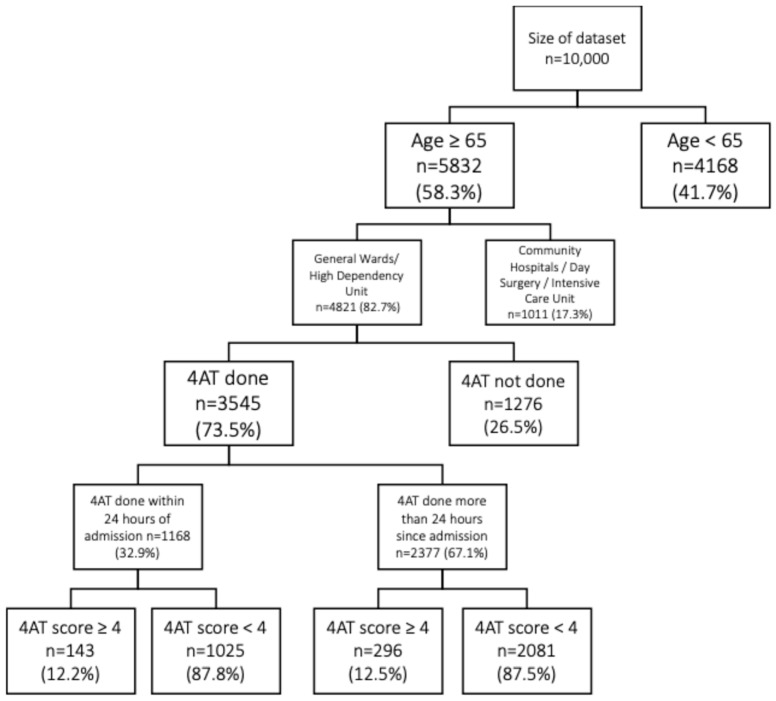
Number of reviewed patients, overall number of patients assessed for 4AT, and number of 4AT done within 24 h of hospital admission.

A total of 4821 patients were eligible for assessment (Figure 1). Of these, 73.5% (*n* = 3545) underwent 4AT assessment. Out of the 3545 individuals, only 32.9% (*n* = 1168) were completed within 24 h of admission. The remaining 67.1% (*n* = 2377) had their 4AT screening after 24 h (Figure 1).

Among patients assessed within 24 h of admission, 12.2% (*n* = 143) scored ≥4 on the 4AT, indicating a potential delirium diagnosis (Figure 1). In comparison, 12.5% (*n* = 296) of patients assessed after 24 h scored ≥4 on the 4AT (Figure 1).

### 3.2. IDC and Timeliness with 4AT Screening

Our descriptive observation found patients scoring 4 and assessed within 24 h of admission had higher use of IDC compared to those assessed after 24 h. The results were found to have a statistically significant association (*p* = 0.01) (Table 2).

### 3.3. Length of Stay and Timeliness with 4AT Screening

Our descriptive observation found a statistically significant association between 4AT timeliness (patients scoring ≥ 4 and 4AT done ≤ 24 h versus >24 h) and the LOS categories (*p* = 0.02).

### 3.4. Physical Restrains Use and Timeliness with 4AT Screening

There was also statistically significant association between 4AT timeliness (patients scoring ≥ 4 and 4AT done ≤ 24 h versus >24 h) and the use of restraints (*p* = 0.03).

## 4. Discussion

### 4.1. Suboptimal Compliance with the 4AT Assessment Protocol

The findings from this study suggest that there has been suboptimal compliance with the 4AT assessment protocol. While the majority of nurses were performing the assessment, the implementation may not be occurring as routinely or systematically as intended to ensure all eligible patients are screened. Consistent with our findings, Sinvani et al. (2016) [16] identified a poor understanding of delirium and its confusion with dementia as common barriers to delirium screening. While delirium is known to be associated with negative outcomes, health professionals’ knowledge about delirium is generally poor [17]. Qian et al. (2025) [18] also mentioned that, alongside limited understanding of delirium among healthcare professionals, difficulties using diverse and often non-standardized assessment tools, high workloads, and insufficient training can contribute as major barriers that prevent effectively screening, recognizing, and managing delirium.

Also, without clear guidelines or institutional mandates for routine delirium screening, healthcare providers may overlook or deprioritize this assessment, particularly in high-pressure or time-sensitive situations. Alhaidari et al. (2022) [13] highlighted one of the main barriers to completing the 4AT was unstructured delirium assessments. Instead of conducting structured assessments, doctors prioritized urgent care and comfort, and relied upon general observations of the patient and clinical gestalt to diagnose or rule out delirium [13]. Additionally, staff workloads and competing priorities, such as emergency interventions or the management of acute conditions, might contribute to delays in or omissions of the 4AT screening, despite its demonstrated importance in early delirium detection [13]. This was reflected by the underdiagnosis of delirium, which has been widely documented in the literature [19,20]. Bianchi et al. (2024) [21] also mentioned that delirium in hospitalized older adults was often inconsistently recognized and managed, leading to potential adverse outcomes.

### 4.2. Timeliness of Performing 4AT Assessment

The findings from this paper indicated that the majority of the 4AT delirium assessments were performed more than 24 h after admission, which raises important concerns about the timeliness of delirium screening practices in clinical settings. This is evident from the literature where there may still be a lack of widespread recognition among certain healthcare professionals regarding its early identification as a critical element in patient management [18]. Coupled with time constraints in busy hospital environments, the 4AT assessment may not be viewed as a priority, leading to inconsistent usage [22]. In addition, due to the nature of delirium often presenting with fluctuating symptoms, it might make timely assessment difficult or be overlooked. Ultimately, improving adherence to the 4AT assessment could play a pivotal role in reducing the burdens of delirium, improving patient outcomes, and minimizing the long-term cognitive and functional impairments often associated with delayed diagnosis and management [23]. Anand et al. (2022) [24] mentioned conducting 4AT within 24 h facilitated early detection and management, which could significantly improve patient prognosis.

### 4.3. Characteristics

#### 4.3.1. Indwelling Catheter Use

Patients who scored ≥4 for their initial assessment and were screened more than 24 h after admission were observed to have a higher percentage of IDC use. Studies showed that delayed assessment can lead to more complex interventions, prolonging delirium episodes [25]. This may be attributed to delirious patients often experiencing cognitive impairment, reduced awareness, and disorientation, which can impair their ability to recognize the need to void or communicate toileting needs, increasing the likelihood of catheter insertion [26,27].

#### 4.3.2. Restraints Use

Patients who scored ≥4 for their initial assessment and were screened more than 24 h after admission were observed to have higher percentage of restraint use. This is consistent with findings reported in the literature where delayed recognition of delirium often resulted in missed opportunities for early intervention [28], leading to the progression of behavioral symptoms and increased reliance on restraints to manage agitation or confusion [29,30]. Delayed assessments can lead to more complex interventions and prolong delirium episodes [25]. Improving compliance with early 4AT screening could serve as a preventive measure, facilitating earlier treatment and potentially reducing the need for restrictive interventions [31].

#### 4.3.3. Length of Stay

Lastly, patients who scored ≥4 for their initial assessment and were screened within 24 h of admission were found to have a shorter length of hospital stay compared to those assessed later. In their study, Artola et al. (2020) [32] demonstrated similar results as early 4AT screening enabled prompt, targeted management of reversible factors which potentially shortened delirium duration. Timely assessments also allowed for early implementation of non-pharmacological bundles, which have been shown to reduce delirium severity and LOS [33]. Also, it has been shown that delayed assessments can lead to more complex interventions, prolonged delirium episodes, and consequently longer hospital stays [34].

### 4.4. Limitations

This study has several limitations that should be considered when interpreting the findings. First, challenges arose with high dependency units (HDUs), where some wards combined general and HDUs under the same unit designation. As a result, we were unable to completely separate data from HDUs, which may have led to heterogeneity within the sample [35]. Patients in HDUs often have more complex medical conditions and higher acuity, which could influence delirium incidence and the compliance with the 4AT protocol [36]. This overlap may have affected the accuracy of the reported compliance rates, as clinical priorities in HDUs differ from those in general wards. Also, as a single-center study, generalizability beyond similar settings should be interpreted cautiously.

The study did not examine reassessment compliance throughout the hospitalization stay. Delirium can fluctuate over time, with patients experiencing periods of improvement or worsening, which can make ongoing assessment critical for adjusting care plans and interventions [37]. This led to recognition of the need for more research in this gap. In addition, analyses were unadjusted. Hence, observed associations should be interpreted as descriptive and not as independent or causal relationships.

Lastly, this paper mainly reported descriptive and unadjusted findings. Logistic regression modeling was not performed. As a result, potential confounding factors could not be fully accounted for. Therefore, observed associations should be interpreted cautiously.

### 4.5. Recommendations

Aligning with broader implementations in the literature, organizations could look into providing supportive strategies to improve delirium detection in hospitals. In their study, Benn et al. (2025) [38] mentioned electronic delirium alert systems and education initiatives for hospitalized older adults as a feasible strategy which can enhance screening. System-level strategies might be necessary to improve delirium detection in acute hospitals. This underscores the importance of organizational support in fostering delirium screening in acute hospitals.

## 5. Conclusions

Through this study, we have distinguished between screening compliance and timeliness. The results from this study have shown that a substantial proportion of older adults inpatients were being screened for delirium risk using the 4AT tool. However, the challenge lies in completing the assessment within the recommended 24 h window. These findings showed that while nurses were using 4AT tool to screen the patients, delays in assessments have persisted, which may have implications on the early management of delirium in older adult patients.

Hence, this study can provide new insights into where further quality improvement and research efforts should be focused or needed. By improving the system around timeliness, we can ensure timely management for high-risk patients and improve safety for our older patients.

## Figures and Tables

**Table 1:** Eligibility Criteria.

Inclusion	Exclusion Criteria
(1) Patient ≥ 65 years old and above	(1) Patients < 65 years old
(2) Admitted to acute ward settings	(2) Admitted to intensive care units, day surgery, discharge lounges, and community hospitals
(3) Admitted during the study period 1 June 2022–30 June 2023	

**Table 2:** Data Characteristics of patients.

	4AT Score ≥ 4 (*n* = 439)		
Patient Characteristics	Patients 4AT Done Within 24 h of Admission	Patients 4AT Done More Than 24 h Since Admission	*p* Value
Total Number	*n* = 143	*n* = 296	
Gender	n (%)	n (%)	
Male	59 (41.3%)	152 (51.4%)	*p* = 0.05
Female	84 (58.7%)	144 (48.6%)	
Ethnicity	n (%)	n (%)	
Chinese	124 (86.7%)	248 (83.8%)	*p* = 0.61
Malay	6 (4.2%)	19 (6.4%)	
Others	13 (9.1%)	29 (9.8%)	
Activity Assessment	n (%)	n (%)	
Bedfast	103 (72.0%)	199 (67.2%)	*p* = 0.69
Chairfast	29 (20.3%)	68 (23.0%)	
Walks frequently	2 (1.4%)	2 (0.7%)	
Walks occasionally	8 (5.6%)	25 (8.4%)	
Blanks	1 (0.7%)	2 (0.7%)	
Indwelling Catheter	n (%)	n (%)	
Yes	54 (37.8%)	150 (50.7%)	*p* = 0.01 *
No/Blanks	89 (62.2%)	146 (49.3%)	
Length of Stay	n (%)	n (%)	
≤5 days	62 (43.4%)	90 (30.4%)	*p* = 0.02 *
6–10 days	37 (25.9%)	79 (26.7%)	
11–15 days	19 (13.3%)	39 (13.2%)	
>15 days	25 (17.5%)	88 (29.7%)	
MUST Score	n (%)	n (%)	
0	39 (27.3%)	84 (28.4%)	*p* = 0.07
1	3 (2.1%)	22 (7.4%)	
≥2	31 (21.7%)	73 (24.7%)	
Blanks	70 (49.0%)	117 (39.5%)	
Restraints	n (%)	n (%)	
Yes	17 (11.9%)	60 (20.3%)	*p* = 0.03 *
No	126 (88.1%)	236 (79.7%)	

MUST: Malnutrition Universal Screening Tool. Categorical variables are presented as n (%). Group differences were assessed using Pearson’s chi-square test or Fisher’s exact test where expected cell counts were <5. Statistical significance was defined as * *p* < 0.05.

## Data Availability

The data are not publicly available due to privacy or ethical restrictions.

## Abbreviations

The following abbreviations are used in this manuscript:

AMT	Abbreviated Mental Test
HDUs	High Dependency Units
IDC	Indwelling Catheter
LOS	Length of Stay
